# Microstructure of Transparent Strontium Fresnoite Glass-Ceramics

**DOI:** 10.1038/srep09069

**Published:** 2015-03-17

**Authors:** Wolfgang Wisniewski, Kazuya Takano, Yoshihiro Takahashi, Takumi Fujiwara, Christian Rüssel

**Affiliations:** 1 Otto-Schott-Institut, Jena University, Fraunhoferstr. 6, 07743 Jena, Germany; 2 Department of Applied Physics, Tohoku University, 6-6-05 Aoba, Aoba-ku, Sendai 980-8579, Japan

## Abstract

Glass-ceramics grown from a glass of the composition Sr_2_TiSi_2.45_O_8.9_ (STS 45) are analyzed by scanning electron microscopy (SEM) and electron backscatter diffraction (EBSD). Oriented nucleation with the c-axes preferably perpendicular to the surface is detected. A very strong 001-texture is observed after only 10 μm of growth into the bulk, making this the first system in which an orientation preferred during nucleation prevails during growth into the bulk in glass-ceramics. Piezoelectric measurements are performed and d_33_-values presented and discussed. The obtained results are critically viewed with respect to the two growth models describing Sr_2_TiSi_2_O_8_ growth in glasses.

The crystallization of fresnoite from glasses leads to challenging materials due to their combination of the piezoelectric, pyroelectric, surface acoustic and nonlinear optical properties. Heat capacity measurements of powdered fresnoite^1^ and fresnoite glass-ceramics were also performed recently^2^. Nano indentation measurements have also been performed on these materials^3^. Since fresnoite is a polar non-ferroelectric crystal phase, macroscopic piezoelectricity can only be achieved by a high degree of orientation. Statistically oriented materials do not show piezoelectric properties^4^. Fresnoite of the composition Ba_2_TiSi_2_O_8_ (BTS) may form solid solutions with the systems Sr_2_TiSi_2_O_8_ (STS, recently studied by synchrotron X-ray powder diffraction^5^,^6^) and Ba_2_TiGe_2_O_8_ where GeO_2_ is used to substitute SiO_2_^7^,^8^,^9^,^10^. While oriented fresnoite has been prepared in the form of thin films^11^,^12^ or thin crystal lines written in a glass matrix by laser induced crystallization^13^,^14^, larger bodies of oriented fresnoite glass-ceramics may be produced by electrochemically induced nucleation^15^ and surface crystallization. While temperature gradients have been applied during the latter in the past^16^,^17^, more recent results have shown that oriented layers of crystals also occur without this experimental effort. The surface crystallization of STS, from glasses shows especially great potential due to a lack of bulk nucleation. This enables to prepare thick layers of oriented crystals^18^,^19^,^20^,^21^,^22^,^23^.

Recently two very different growth mechanisms have been formulated for the growth of STS in two different glass compositions: glass A of the composition STS+0.45 SiO_2_ (Sr_2_TiSi_2.45_O_8.9_) is being studied in Japan with a focus on preparing transparent glass-ceramics for optical applications^18^,^19^,^20^,^21^ while glass B of the composition STS+0.75 SiO_2_ (Sr_2_TiSi_2.75_O_8.5_) is being studied in Germany. This glass-ceramic was designed with a focus on the piezoelectric properties. Furthermore, fundamental studies were carried out concerning crystal nucleation and the understanding of oriented nucleation and crystal growth in glasses^22^,^23^,^24^. In both glasses, SiO_2_ has been added to the quickly crystallizing stoichiometric system in order to decrease the crystallization tendency of the glass. In this system, the only crystallizing phase is stoichiometric fresnoite. Hence, the excess SiO_2_ occurs in the form of a SiO_2_-enriched residual glassy phase after crystallization.

The growth model A was proposed for glass A, is formulated in Ref. 21 and is based on a metastable immiscibility in the glass which leads to the formation of SiO_2_-rich nano droplets embedded in a strontium and titanium rich matrix above the glass transition temperature T_g_. The formation of the droplet phase shifts the matrix composition towards the stoichiometry of STS. This in turn facilitates the crystallization of fresnoite via inhomogeneous nucleation at the sample surface. The SiO_2_ nano particles are captured at the fresnoite growth front and hence frozen into the crystallizing matrix. This growth model has also been suggested to apply for a glass of the composition BTS+0.54 SiO_2_ where similar results have been obtained^25^. In both systems, a 001 texture is observed which is assumed to result from a statistical nucleation followed by a kinetic selection during growth^25^. A statement concerning the speed of growth selection has not been published so far.

The growth model B was proposed for glass B, is formulated in Ref. 22 and is based on oriented nucleation at the surface where a 001 texture is observed in discrete crystals. During growth into the bulk, these crystals fray into structures which show increasingly diverse orientations where the fastest average direction of crystal growth is with the c-axes of the crystals tilted by about 43° to the main growth direction. A corresponding texture is observed 300 μm below the surface after a relatively slow growth selection. This texture is proposed to be caused by the local buildup of residual SiO_2_ in the fastest growing 001 direction which may be circumvented by the proposed growth mechanism. The bulk microstructure of this material is described as a kind of crystalline sponge highly permeated by channels of residual glass without any discernible grain boundaries and has been proposed to possibly be the result of a growth mechanism called viscous fingering^24^.

Both systems share the observation of a 001 texture and no sign of an SiO_2_ enriched diffusion layer at the growth front. Both systems are transparent after short annealing times but become opaque if annealing proceeds at high temperatures for longer times^18^,^22^. This last aspect indicates that the microstructure is not stable after the crystallization front has passed but instead the crystal domains continue to grow via diffusion until they become large enough to interfere with light of visible wavelengths. Both of these effects may be explained by the respective growth models^21^,^22^.

There are two central aspects to note concerning the growth model proposed to occur in glass A: i) in contrast to the proposed statistically oriented nucleation, a preferred orientation of the formed nuclei has been proven to occur in STS^22^,^23^ and BTS^26^,^27^ using glasses containing a higher SiO_2_ concentration. So far EBSD is the only method with which oriented nucleation may be detected with certainty and EBSD has not been applied to the systems for which the growth model A is proposed to be valid. ii) High quality TEM results of glass A featuring a single crystal domain at the surface as well as the growth front only show uncrystallized glass, crystalline fresnoite and residual glass^19^. An area where two phase separated liquids coexist was not proved. While this aspect raises the question whether the proposed growth model is applicable, the observed results may e.g. be explained by crystallization proceeding during cooling after the phase separation no longer occurs. Hence the crystallization front could catch up to the phase separation front during cooling. Additionally, the question arises why there is no nucleation at the liquid – liquid interface after phase separation and solely surface nucleation is observed instead.

The glasses A and B are produced quite differently. Glass A is produced by melting the reagents in an electric furnace and splat cooling the melt on a stainless steel plate preheated to 220°C to produce glass plates of 1 mm thickness^18^. The production of glass B allows samples with a thickness of at least 5 mm and is achieved by melting the reagents in an inductively heated furnace, poring the melt onto a brass block, quenching it with a stamp for ca. 7 s and subsequently transferring the batch to a furnace preheated to 790°C where the glass is then cooled slowly^22^.

The aim of this article is to clarify the discrepancies between the published growth models by applying EBSD to the glass A and gaining insight into the nucleation and orientation development of STS during growth in this system.

## Results

As STS glass-ceramics grown from a number of compositions have been featured in numerous articles, the experiments performed for this article are also aimed at confirming the comparability of samples prepared from the same glass but by different groups. For this aspect, some samples were polished and annealed in Japan to ensure comparability with previous preparations while other samples were polished and annealed in Germany to test the reproducibility of the results when using different experimental environments, e.g. different furnaces and polishing procedures.

**Figure 1:**
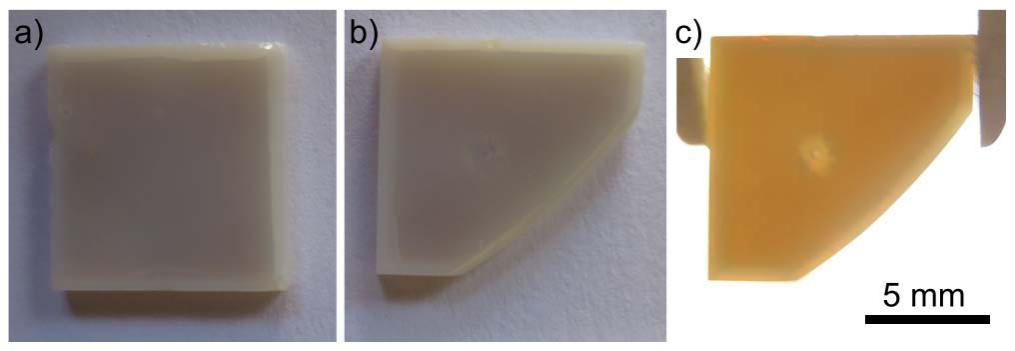
Photographs of 1 mm thick STS45 samples annealed for 20 h at 970°C.

Samples of glass A were annealed at 970°C for 20 h to produce comparable results to those obtained from the glass B^22^. Photographs of the precursor glass and a transparent sample have been published in Ref. 21. In contrast to the transparent glass ceramics previously achieved by annealing at lower temperatures for shorter time periods^18^,^19^,^20^,^21^, the resulting samples were rather opaque as shown in Figure 1a) and b). This is in agreement with the bluish white color observed after annealing at 880°C^18^. As these samples are only 1 mm thick, they are somewhat translucent if held against a strong light source, compare Figure 1b) and c) which feature the same sample. The bright ring at the edges illustrates the area where three crystallization fronts interact due to the surface crystallization initiated from the sides of the samples.

**Figure 2:**
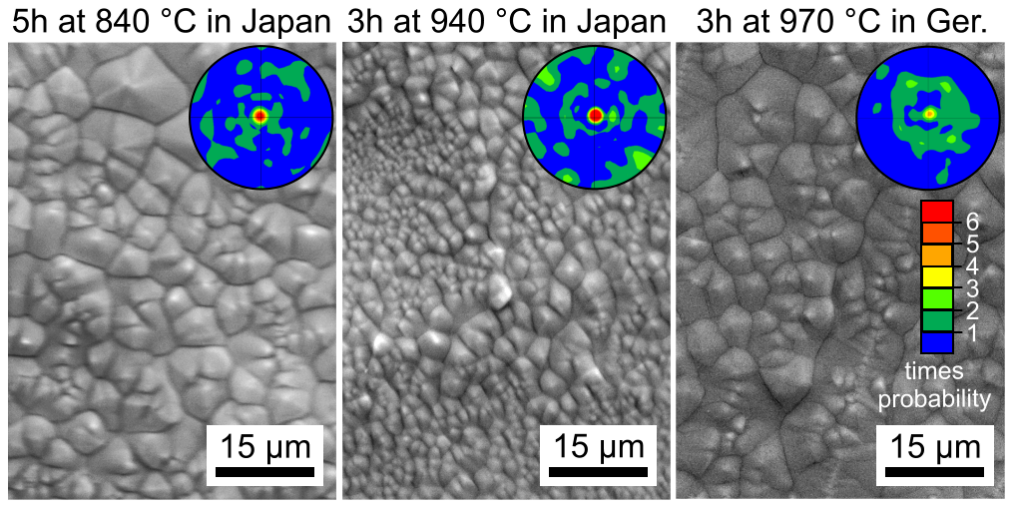
SEM-micrographs and 001 pole figures of textures calculated from EBSD-scans illustrating the crystal morphology and preferred crystal orientations at the immediate surfaces of STS45 samples annealed under the stated circumstances.

Figure 2 features SEM-micrographs obtained from samples of glass A polished and annealed for different times and temperatures (see figure captions). Some of the samples were annealed in Germany, others in Japan. Due to the short annealing times, these samples were transparent in agreement with the literature^20^,^21^. These micrographs illustrate the surface morphology resulting from three annealing temperatures and times for samples placed into preheated furnaces in order to minimize the nucleation time. They present surface morphologies featuring individual crystals which are quite similar to the surface crystals observed in glass B^22^,^23^. EBSD-scans performed on these surfaces show that each crystal is indeed a single crystal and shows a single homogeneous orientation while a systematic localization of any specific orientation to a discernible area of crystallization is not observed. The superimposed 001 pole figures (PFs) of textures calculated from these EBSD-scans show 001 textures for every surface, relatively weak in the sample prepared in Germany, implying oriented nucleation in glass B with the c-axes preferably oriented perpendicular to the sample surface. Please note that the cause of oriented nucleation in glasses during surface crystallization is still unknown, making any discussion of the texture intensities very speculative.

**Figure 3:**
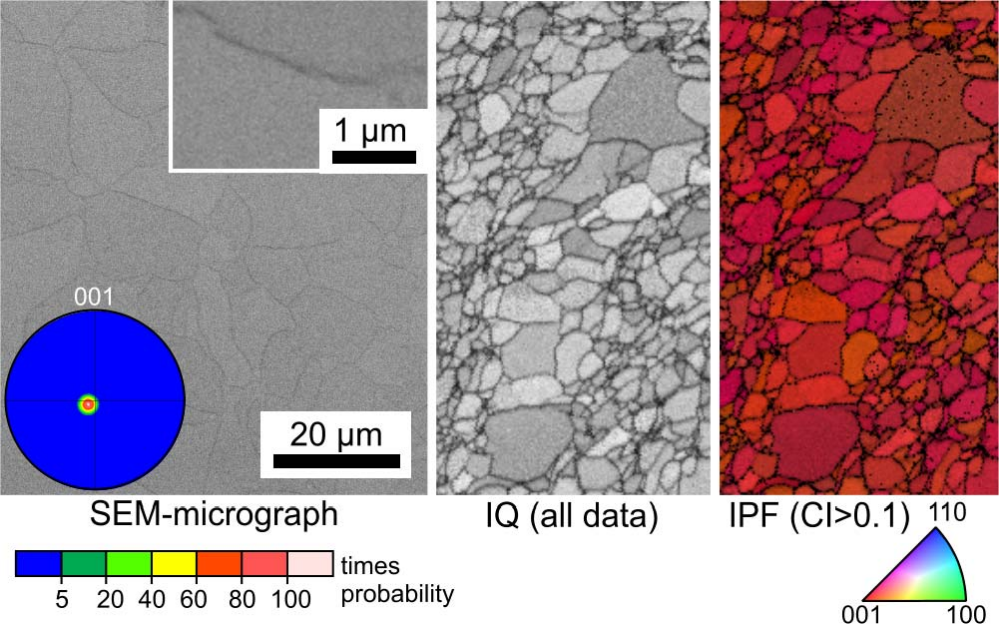
SEM-micrographs of the microstructure after removing 100 μm from the surface of a transparent sample annealed at 940°C for 3 h. The IQ-map and IPF-map of a performed EBSD-scan are presented along with the 001-PF of a texture calculated from the scan.

**Figure 4:**
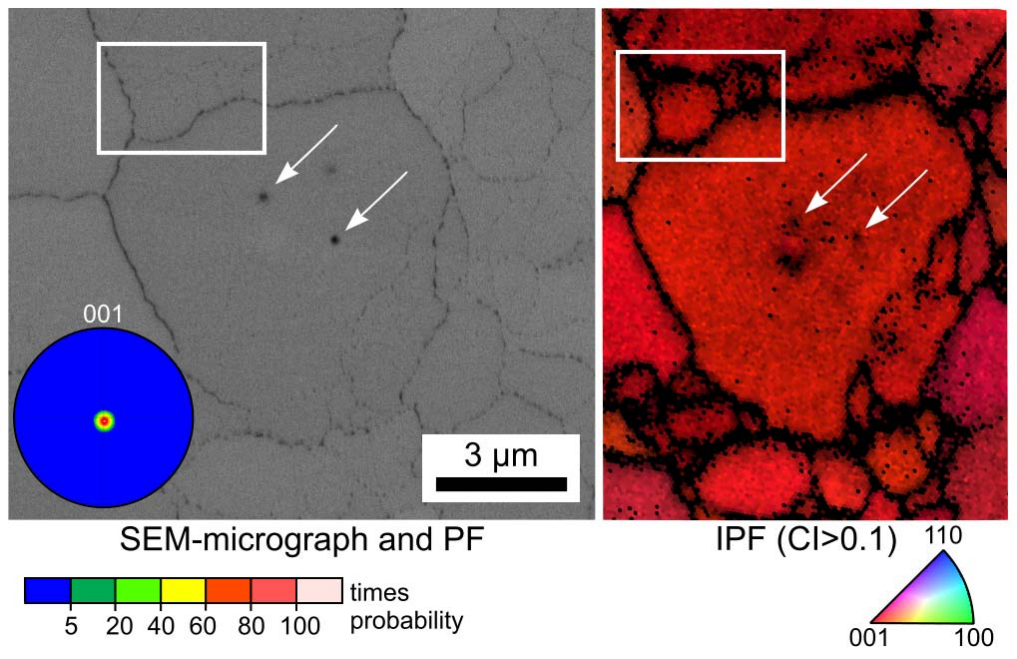
SEM-micrograph of the microstructure of an opaque sample annealed at 970°C for 20 h. The IPF-map of a performed EBSD-scan is presented along with the 001-PF of a texture calculated from the scan.

The microstructures of cut planes parallel to the surface and ca. 100 μm beneath the latter are illustrated in the Figures 3 and 4. Figure 3 presents results from a completely crystallized, transparent sample annealed at 940°C for 3 h. The SEM-micrograph shows barely discernible pseudo grain boundaries between crystallized areas which do not show discernible inclusions of residual SiO_2_ comparable to those observed in the glass B^22^ as highlighted by the inset. The term “pseudo grain boundaries” is used because the term “grain boundaries” is not commonly used to describe an area enriched in residual glassy phase. These boundaries are clearly discernible in the image quality-map (IQ-map) of a performed EBSD-scan and the corresponding inverse pole figure map (IPF-map) shows that all data points are 001 oriented in this cut plane. The 001-PF of a texture calculated from this scan confirms this impression by an extreme 001 texture where orientations with the c-axes perpendicular to the surface occur with a probability of more than 100 times the probability in a statistical distribution.

The comparable results of the opaque sample annealed for 20 h at 970°C and presented in Figure 1a) are presented in Figure 4. The SEM-micrograph shows clearly discernible pseudo grain boundaries and some inclusions which may be assumed to be residual SiO_2_, see arrows. They are not pores due to the lack of an edge effect in the SEM-micrograph. Both features are also observed in the IPF-map of an EBSD-scan performed on the area with a step size of 100 nm. The frame highlights an area where only two pseudo grain boundaries are clearly observed in the SEM-micrograph while at least three are implied by EBSD. As in Figure 3 it is clear that the pseudo grain boundaries affect the EBSD-analyses much more than the SEM-micrograph which may be explained by an accumulation of amorphous residual SiO_2_ in these areas. A strong 001 texture obtained from a larger scan of 156 × 394 μm^2^ is also presented.

**Figure 5:**
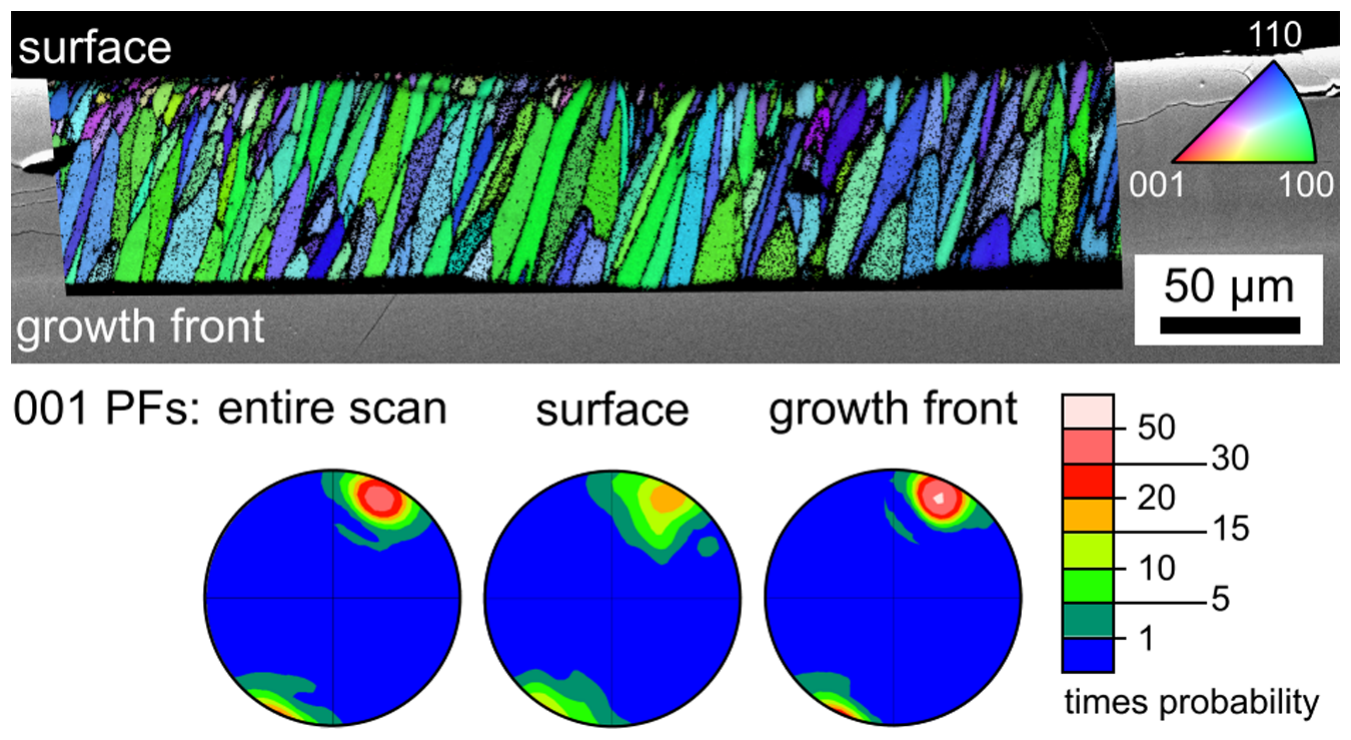
SEM-micrograph of the surface crystallized layer in a cross section of a transparent sample annealed at 840°C for 5 h superimposed by the IPF+IQ-map of an EBSD-scan. 001-PFs calculated from the entire scan data, the surface layer and the growth front are presented below.

Cross sections were prepared in order to visualize the crystal growth into the bulk. Figure 5 presents an SEM-micrograph superimposed by the IPF+IQ-map of an EBSD-scan performed on the cross section of a sample annealed at 840°C for 5 h. This transparent sample shows a crystal layer of about 80 μm around a core of uncrystallized glass. The figure shows that a relatively fast kinetic selection occurs within the first ca. 10 μm of growth: the number of growing crystals clearly decreases just below the surface when comparing Figure 5 to the surfaces featured in Figure 2. After this initial selection, further growth selection is limited and a relatively even growth front is observed. The 001-PFs presented below verify the kinetic selection: close to the surface, the texture is much less discrete than at the growth front or compared to the entire scan. Channels of residual SiO_2_ oriented parallel to the growth direction comparable to those observed in glass B could not be detected by SEM.

**Figure 6:**
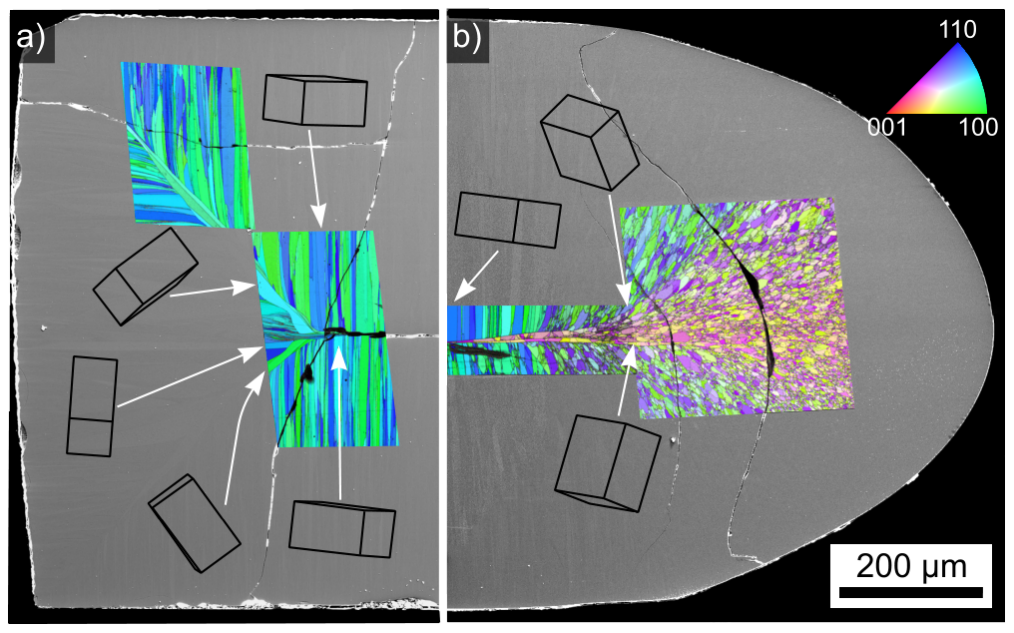
SEM-micrographs of the cross section of a sample annealed at 970°C for 20 h superimposed by IPF-maps of performed EBSD-scans: a) rectangular corners and b) a rounded edge. Wire frames of unit cells visualize specific orientations at the respective locations.

Opaque samples annealed at 970°C for 20 h are completely crystallized and the respective crystallization fronts have collided due to the lack of nucleation in the bulk. Figure 6 shows two cases of crystallization: a) were three crystallization fronts start at three rectangular sides of the sample and b) were the sample is rounded and hence a radius of surface normals is observed. Neither the fragmentation of homogeneously oriented areas nor dendritic growth occur. However, the growth front interaction described for glass B^22^ is also observed here: the growth velocity is increased in the areas where the respective thermal fields overlap and the crystals change their direction of growth without changing their orientation. In fact, Figure 6a) actually shows grains of STS oriented with their c-axes tilted by 45° to the grains oriented perpendicular to their respective surfaces growing between the neighboring growth fronts, which is likely a special case of growth selection for this locality. However, these crystals do not collide in the current cut plane but are blocked by a spike of perpendicular growth from the left side which grew even faster due to the local velocity acceleration^22^. This spike is extremely pronounced at the radial side of the sample featured in Figure 6b).

Finally the method of EBSD-pattern degradation^28^ was applied to unpolished and polished surfaces of samples annealed at 970°C for 20 h. In both cases comparable results were obtained for step sizes down to 10 nm which is in agreement with the results obtained from glass B^22^. Total pattern degradation could not be achieved and a glass layer covering the crystals was not detected.

In order to compare the piezoelectric activity of the glass-ceramics produced from glass A, 0.5 mm thick samples were prepared from the transparent and the opaque glass-ceramics and d_33_-values were determined. The d_33_-value illustrates the piezoelectricity and shows the degree of polarity. Surprisingly, the average d_33_-values were only 6.95 pC*N^−1^ (transparent) and 2.05 pC*N^−1^ (opaque) which is in contrast to the high values obtained from glass B where an average value of 10.8 pC*N^−1^ was measured^23^. This is surprising because of the high degree of crystal orientation and the lower concentration of residual glassy phase in glass A. Furthermore, the alignment of the crystallographic c-axes is much more suited to maximize the piezoelectric effect than the orientation of the c-axis in an angle of 43° observed in glass B at some distance from the surface^22^.

The easiest explanation for the low d_33_-values would be an imperfect sample preparation: as the initial samples are only 1 mm thick and only half the thickness can be used to measure the piezoelectricity of one layer of crystallization, it is quite possible that some of the crystal growth from the opposite side was not removed from the samples which were produced by leveling the surface, then flipping the samples over and grinding them to a thickness of 0.5 mm before contacting them with gold.

Assuming the d_33_-samples contain only crystals from one layer of crystallization, the ability to measure d_33_-values could be caused by a preference of either 001 or 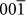 oriented crystals during growth as this would reduce the overall piezo-electric effect. However, we cannot distinguish a 001 from a 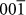 orientation (opposite polarities) in this system using EBSD at this point due to pseudosymmetry effects.

## Discussion

In summary, the oriented nucleation already described for STS in Glass B^22^, BTS^26^, diopside^29^ and BaAl_2_B_2_O_7_ crystals in glass ceramics^30^ was also detected in glass A after annealing at temperatures of 840, 940 and 970°C. Hence the detected 001 preference occurs over a wide range of temperatures similar to the 101 texture observed in BTS where additional textures were observed as the annealing temperatures were increased^27^. However, in contrast to the previous cases where the orientation preferred during nucleation never prevailed during growth, the 001 orientation observed at the surface of glass A quickly dominates the microstructure after a growth selection of less than 10 μm, making this the first case where the textures preferred during nucleation and growth are the same.

The different microstructures observed in transparent and opaque samples explains the difference in transparency: while residual SiO_2_ is very finely distributed in the crystallized domains of the transparent samples, inclusions of SiO_2_ and significantly pronounced pseudo grain boundaries enriched in SiO_2_ are detected in the opaque samples. This means the residual SiO_2_ initially remains distributed in between the crystal structure as a phase separating the polar crystals from each other before accumulating into larger aggregates during longer annealing. Hence light scattering is initially minimized because the SiO_2_ domains between the crystals remain very small. On the other hand, the TEM-micrographs of this microstructure^19^,^21^ show that the crystal domains are also very small. Hence it seems quite possible that the transparency observed here is initially similar to the transparency of glass-ceramics where the crystallite size is specifically controlled^31^. During subsequent annealing the samples stay transparent for a time because the SiO_2_ agglomerates in the form of very thin grain boundaries between crystal domains in the μm-scale, perhaps similar to those observed in Sr_3_Al_2_O_6_ ceramics crystallized from glasses^32^. Highly transparent ceramics may also be synthesized by crystallizing stoichiometric glasses of the compositions Sr_1+x/2_Al_2+x_Si_2−x_O_8_ with 0 < x < 0.4 where residual glass was not detected^33^.

With respect to the growth models published in Refs. 21 and 22, the observed growth in glass A is clearly different from that observed in glass B. While the assumption of statistically oriented nucleation^25^ is incorrect, growth into the bulk by viscose fingering would only be possible if the resulting elongated SiO_2_-channels were thin enough so that they would separate due to Rayleigh instabilities and form spherical droplets of SiO_2_ as described in this system by TEM analyses^19^,^21^. The phase separation used as a basis for growth model A^21^ remains unproven experimentally and the question why there is no nucleation at the liquid-liquid interface after a possible phase separation also remains unsolved. The observed pseudo grain boundaries could be explained in both growth models: if crystal domains from the surface grow through a phase separated region, their boundaries may be locations preferred for the accumulation of residual SiO_2_. On the other hand, neighbouring domains of separately growing viscose fingers could interact in the same way.

The low d_33_-values measured for glass A are quite surprising because the crystal alignment is much more suited for high d_33_-values than that of glass B. These low values are possibly the result of the very thin samples produced from the 1 mm thick glass sheets currently available. Thicker samples would be preferred and perhaps lead to different results, but producing thicker samples using the applied method is not trivial.

## Methods

1 mm thick samples of glass A were produced in Japan according to the method outlined in Ref. 18 and subsequently sent to Germany for EBSD analysis. In order to eliminate an effect of the polishing procedure, annealing programs and furnaces, some samples were polished and annealed in Japan while others were polished and annealed in Germany using the polishing procedure outlined in Ref. 22 directly before analysis. The samples annealed in Japan were placed into the furnace at room temperature while the samples annealed in Germany were transferred into a furnace preheated to the annealing temperatures ranging from 840 to 970°C. They were annealed for up to 20 h in order to grow STS crystals and allow comparability to previous investigations^21^,^22^.

All samples for scanning electron microscopy (SEM) were contacted with Ag-paste and coated with a thin layer of carbon at about 10^−3^ Pa to avoid surface charging. The samples were analyzed using a Jeol JSM-7001F scanning electron microscope equipped with an EDAX Trident analyzing system containing a TSL Digiview 3 EBSD-camera. EBSD-scans were captured and evaluated using the programs TSL OIM Data Collection 5.31 and TSL OIM Analysis 6.2. The scans were performed using a current of about 2.40 nA (measured with a Faraday cup) and a voltage of 20 kV. Only points with a minimum Confidence Index (CI)^34^ of 0.1 were considered in EBSD-maps indicating the attributed orientation solutions are correct with a probability of at least 96%.

The method of EBSD-pattern degradation was applied to the material using the same experimental conditions stated in Ref. 29 to ensure comparability: 20 kV, 4.55 nA, binning: 2 × 2, gain: 0, exposure: 0.45 s and 50 steps per row. As in all recent experiments to this topic, a digital filter composed of 1: background subtraction (Avg. 10), 2: mean smoothing filter, 3: dynamic background subtraction (passes: 20, balance: 100) and 4: normalize intensity histogram was applied during EBSD-pattern acquisition.

Samples for d_33_-measurements were produced by leveling the surface of 1 mm thick crystallized samples, flipping the samples over and grinding them to a thickness of 0.5 mm and finally contacting them with gold via sputtering. The d_33_-values were measured using a d_33_-meter (Sinocera YE2730A), see also Ref. 23.
